# Intentional gestural communication amongst red-capped mangabeys (*Cercocebus torquatus*)

**DOI:** 10.1007/s10071-022-01615-7

**Published:** 2022-04-01

**Authors:** Anne Marijke Schel, Axelle Bono, Juliette Aychet, Simone Pika, Alban Lemasson

**Affiliations:** 1 Animal Behaviour and Cognition, Utrecht University, Padualaan 8, 3584 CH Utrecht, The Netherlands; 2 Université de Rennes, Normandie Université, CNRS, EthoS (Éthologie Animale et Humaine), UMR 6552, 35000 Rennes, France; 3 Department of Ecology and Evolution, Universite de Lausanne, Biophore, 1015 Lausanne, Switzerland; 4 Comparative BioCognition, Institute of Cognitive Science, University of Osnabrück, 49076 Osnabrück, Germany

**Keywords:** Gestural communication, Intentionality, Flexibility, Primates, Monkey, Manual

## Abstract

Apes, human’s closest living relatives, are renowned for their intentional and highly flexible use of gestural communication. In stark contrast, evidence for flexible and intentional gestural communication in monkeys is scarce. Here, we investigated the intentionality and flexibility of spontaneous gesture use in red-capped mangabeys (*Cercocebus torquatus*). We applied established methods used in ape gesture research to analyse whether the body acts produced by a total of 17 individuals living in three different groups in captivity qualified as intentionally produced gesture instances. Results showed that signallers showed all hallmarks of intentionality during the production of 20 out of a total of 21 different types of body acts. These were only produced in the presence of other individuals, and the monkeys showed audience checking, sensitivity to the attentional states of recipients, adjustment of signal modality, and response waiting relative to their production. Moreover, in case of communication failure, the monkeys showed goal persistence, and regarding the production contexts they showed some signs of means–ends dissociation. Therefore, these monkeys are capable of flexible and intentional gestural communication and use this to communicate with conspecifics. Our results corroborate recent findings showing that intentional gestural communication was already present in the monkey lineage of catarrhine primates. We discuss our results in light of the comparative approach towards human language evolution and highlight our finding that these monkeys also showed flexible and intentional use of four ‘free’ manual gesture types.

## Introduction

Human language is a unique communication system in the animal kingdom, which crucially depends on a complex interplay of several sensory–motor, cognitive and computational capacities (Hauser et al. 2002). Trying to unravel its evolutionary roots has been a primary interest of researchers studying non-human animal communication and human language evolution in past decades (e.g. Hewes 1973; Snowdon et al. 1982; Seyfarth 2005; Arbib et al. 2008; Tomasello 2010; Petkov and Jarvis 2012; Lemasson et al. 2013; Fitch 2017). In this context, past comparative research revealed that precursors or pre-adaptations for several cognitive abilities needed for language can be found in non-human animal communication systems (Hockett 1959; Fitch 2010; Hauser et al. 2002). One of these is the ability to communicate intentionally, which requires from individuals involved in the communicative act a certain degree of intentionality. In cognitive and philosophical sciences, intentionality has been described as the capacity of mental states (e.g. knowing, believing and desiring) to be ‘about’ objects, properties, or situations (including other mental states), or to ‘refer’ to them (reviewed in Searle 1983; Dennett 1983). Although originally a purely philosophical concept considered only applicable to mental phenomena, it has subsequently been applied to explain the intentionality of behavioural acts, such as communicative behaviour in humans (Grice 1957) and animals (Premack and Woodruff 1978; Dennett 1983).

In particular, Dennett’s (1983) framework for considering the degree of intentionality underlying animal behaviour has become the topic of reheated interest and debates in recent comparative communication studies (see, e.g. Scott-Philips 2015; Moore 2016; Fischer and Price 2016; Townsend et al. 2017; Graham et al. 2020; Ben Mocha and Burkart 2021). According to this framework, different levels of intentionality may underlie animal communicative behaviour, ranging from communication with ‘zero-order intentionality’ at one extreme, to overtly intentional or ‘ostensive’ communication at the other. In communication with zero-order intentionality, any form of information transfer between a signaller and a recipient occurs as a result of the signaller’s and recipient’s automated responses to environmental cues. For this, the individuals neither need to have a mental state of their own, nor to recognise the consequences of their behaviour on the behaviour or mental state of others, i.e. no sophisticated mental processes are required. In stark contrast, for ostensive communication complex mental processes are required, with both signaller and recipient recognising each other’s mental states and behaving accordingly (Grice 1957; Dennett 1983; Sperber and Wilson 1995; Scott-Philips 2015). This latter type has traditionally been regarded as ‘true communication’ and is common in human language (Grice 1957).

The aforementioned scientific debate in comparative communication studies revolves around questions related to the attribution of mental states to animals, i.e. whether any non-human animal has mental states at all, whether they can know them, share them, or manipulate them, whether and how it is possible to measure them, and whether animals would actually need (mutual) mind-reading for successful communicative exchanges (Scott-Philips 2015; Fischer and Price 2016; Moore 2016; Fitch 2016; Townsend et al. 2017; Tomasello and Call 2019; Leavens et al. 2019). Yet, at the same time, non-human animals are increasingly deemed capable of more than mere automated communicative behaviour, with in particular apes having been widely acknowledged to use a form of communication that lies somewhere in between zero-order intentional and ostensive communication, i.e. ‘first-order intentional communication’ (Dennett 1983; Call and Tomasello 2007). This is voluntary (rather than automated) and socially directed (rather than indiscriminately broadcast) communicative behaviour that is produced in a goal-directed way, i.e. with the explicit goal of changing a recipient’s behaviour in a particular way (Dennett 1983; Tomasello 2010; Townsend et al. 2017; Byrne et al. 2017). It is the voluntary and socially directed production that defines this behaviour as deliberately communicative, and that discriminates it from mere informative behaviour that may convey messages without being voluntarily emitted by the signaller to anyone in particular. The goal-directed production, in addition, may reveal something more about the signaller’s goals and intentions underlying its behaviour and its ability to recognise the effects of its own behaviour on the behaviour of other individuals. Following Dennett (1983), these combined capacities could be interpreted as the signaller having a mental state of its own, making it a ‘first-order intentional system’ that is communicating with first-order intentionality.

As mentioned, however, in most animal communication studies it has been common to remain more conservative in interpreting animal communicative behaviour in terms of underlying mental states. Instead, an animal’s deliberate and goal-directed production of communicative acts is often interpreted in leaner ways, e.g. in terms of their apparent ultimate function in relation to the behavioural context in which they occur, or in terms of the ‘apparently satisfactory outcomes’ (ASO’s) of the signalling behaviour that can be determined from the behavioural interplay between signallers and their recipients (Genty et al. 2009; Cartmill and Byrne 2010; Hobaiter and Byrne 2014; 2017; Scott-Phillips 2015; Byrne et al. 2017; Molesti et al. 2020). The latter is considered to be a purely behavioural proxy for the signaller’s ‘intended meaning’ (Genty et al. 2009; Cartmill and Byrne 2010; Hobaiter and Byrne 2014, 2017). Hence, if anything were to be determined at all about potential mental states underlying the animals’ communicative behaviour from such studies, it can at most be concluded that the signaller *behaves as if* communicating with a particular goal in mind (Scott-Phillips 2015; Fischer and Price 2016; Hobaiter and Byrne 2017; Leavens et al. 2019).

Similarly, the methodology used to distinguish between communication with first-order intentionality and mere automated forms of communication has also been designed to largely avoid the question of mental state attribution, and is based on pure behavioural proxies that, across scientific disciplines, have been agreed to be indicative of a signaller’s communicative and goal-directed intentions during social interactions. These behavioural proxies (‘markers’) were originally defined in child development studies investigating the intentionality of non-verbal communication in pre-linguistic children (Bates et al. 1975) and were later adapted to be used in primate research (Tomasello et al. 1994, 1997; Pika et al. 2003; Leavens et al. 2004, 2005; Call and Tomasello 2007; Townsend et al. 2017). Following these definitions, to determine whether or not a communicative event between a signaller and recipient is the product of an intention on behalf of the signaller, it first of all needs to be assessed if a signaller’s behaviour is produced in a voluntary and socially directed way that is conducive to successful communication. This is the case if the behaviour is produced in the presence rather than absence of an audience, is preceded by audience checking (signaller monitors its audience before performing its behaviour and orients to a recipient), and is associated with a signaller’s sensitivity to the attentional state of its recipient (signaller takes into account whether the recipient can perceive its behaviour and adjusts its behaviour if it cannot). Evidence that the behaviour is produced in a goal-directed manner is provided when the signaller shows signs of response waiting after producing it (signaller pauses its behaviour for a given period of time, during which he maintains visual contact, apparently monitoring the recipient’s behaviour), and signs of goal persistence, a flexible behavioural strategy that is contingent on the recipient’s response. Hence, in cases where a signaller’s apparent goal, i.e. a particular behaviour change from the recipient, has been reached it will stop further signalling behaviour, whereas in cases where this goal has not yet been reached, the signaller will perform further signalling behaviour. Furthermore, the use of ‘means–ends dissociation’ (Bruner 1981), in which one signal type is used flexibly in several behavioural contexts and/or several signal types are used in the same context interchangeably towards the same end, is often taken as complementary evidence that the communication is goal directed and intentional (Plooi 1978, 1984; Tomasello et al. 1985, 1997; Liebal et al. 2004; Pika et al. 2003, 2005a; Cartmill and Maestripieri 2012; Fischer and Price 2016; Molesti et al. 2020). And, finally, the use of gaze alternation (signaller alternates its gaze between the recipient and an external object or event of interest) during signal production is considered evidence that the signaller is communicating ‘about’ something to its recipient (Bard 1992; Leavens and Hopkins 1998, Leavens et al. 2004; 2005; Bard et al. 2014; but see Tomasello and Call 2019 for a critical account about this). Together, these ‘markers of intentionality’, thus, specifically focus on assessing both the communicative and goal-directed aspects of an animal’s behavioural acts, with a particular focus on the signallers’ flexibility in obtaining their communicative goals.

Systematic assessment of animals’ use of these markers of intentionality underlying their communicative behaviour has, so far, revealed that the gesture production of all ape species can be qualified as first-order intentional communication (reviewed, e.g. in Byrne et al. 2017; Tomasello and Call 2019; see Schel et al. 2013 for the first evidence of intentional use of vocal communication in chimpanzees). Gestures are a subset of communicative signals that have been defined as recipient-directed movements of the hands, feet, limbs, head, or body postures, which are mechanically ineffective (i.e. they are not designed to act as direct physical agents) and result in a voluntary response from the recipient (e.g. Plooij 1978; Pika 2008; Call and Tomasello 2007; Pika and Liebal 2012; Liebal and Call 2012). The a priori element of recipient directedness defining a gesture already distinguishes gestures from merely automated and indiscriminately broadcast body acts and classifies them per definition as deliberately communicative behaviour. Hence, ape gestures are produced in the presence of an audience (Call and Tomasello 1994; Hostetter et al. 2001; Leavens et al. 2004), with their production generally being preceded by audience checking (Genty et al. 2009; Graham et al. 2017; Hobaiter and Byrne 2011), and signallers showing a sensitivity to the attentional state of their recipient when producing their gestures (Call and Tomasello 1994; Hostetter et al. 2001; Leavens et al. 2004; Pika et al. 2005b; Liebal et al. 2006; Genty et al. 2009). Furthermore, ape gesture production is goal directed, with signallers consistently showing response waiting after producing their gestures (Genty et al. 2009; Hobaiter and Byrne 2011) and clear signs of goal persistence in cases where the presumed communicative goal was not met (Cartmill and Byrne 2007; Leavens et al. 2005; Roberts et al. 2014). Moreover, apes have been found to show flexibility in their gesture production in terms of ‘means–ends dissociation’ (Tomasello et al. 1994; Pika et al. 2005b; Liebal et al. 2006; Genty et al. 2009; Graham et al. 2017), as well as gaze alternation in combination with gesture use in cases where a third entity was involved in the communicative act (Bard 1992; Leavens and Hopkins 1998). These findings have made apes’ gestural communication excellent examples of first-order intentional communication in the animal kingdom, which, subsequently, have been exerting a strong and continued influence on theories on human language evolution (e.g. Arbib et al. 2008; Tomasello and Call 2019; Ben Mocha and Burkart 2021).

In fact, the continued focus on, and fine-tuning of, ape gesture studies has led to some increasingly specific ideas concerning their merit for theories on human language origins (see Cartmill and Hobaiter 2019; Leavens et al. 2019 for discussions about this). An example comes from ape studies focussing specifically on ‘free’ brachio-manual gestures, i.e. gestures only produced with the hands, feet or limbs, without making contact with a substrate or partner (Pollick and de Waal 2007; de Waal and Pollick 2011; Roberts et al. 2012, 2014, 2019). Because of the myriad of contexts the hands are generally used in, free manual gesture production has been considered to be even more flexible than the production of other gesture types (Call and Tomasello 2007). This flexible use of free manual gestures in the apes, in combination with a reported ‘virtual absence’ of such gesture production in monkey species (de Waal 2003; Pollick and de Waal 2007; de Waal and Pollick 2011; Cartmill and Maestripieri 2012; Roberts et al. 2014), has been taken to suggest that the ‘shift toward a more flexible and intentional communicative strategy’ in our ancestors occurred relatively recent, i.e. in the hominoid lineage (sensu Pollick and de Waal 2007; de Waal 2003). However, the necessary comparative work on monkeys’ intentional and manual gesture use to support this idea is currently still largely lacking. To gain a full understanding of both the occurrence and form of intentional gestural communication in the primate lineage and its potential implications for theories on language origins, an equally fine-grained and systematic investigation of monkeys’ gestural communication is needed. The main objective of our study was, therefore, to investigate if monkeys make use of similar forms of intentional gestural communication as found in the apes.

In this respect, several previous studies have already focused on monkeys’ gesture production in experimental setups. These studies showed that some monkeys, like apes, adjust their trained manual gesture production to the presence (*Cercocebus torquatus:* Aychet et al. 2020*)* and attentional states of human experimenters (*Cebus apella*: Hattori et al. 2007, 2010; Defolie et al. 2015; *Saimiri sciureus*: Anderson et al. 2010; *Cercocebus torquatus*: Maille et al. 2012; Aychet et al. 2020; *Papio anubis*: Meunier et al. 2013; Bourjade et al. 2014; Molesti et al. 2020; *Macaca tonkeana*: Canteloup et al. 2015a; *Macaca mulatta*: Canteloup et al. 2015b), indicating the signallers’ communicative intent. Some of these studies also provided evidence for the use of gaze alternation during gesture production (Anderson et al. 2010; Meunier et al. 2013; Bourjade et al. 2014; Canteloup et al. 2015b; Aychet et al. 2020), suggesting that signallers were communicating ‘about’ something to their recipients. Yet, only few studies have focussed on the flexibility of gesture use in terms of goal persistence (*Macaca radiata*: Gupta and Sinha 2019; *Cercocebus torquatus*: Aychet et al. 2020) and means–ends dissociation (*Macaca nemenstrina*: Maestripieri 1996; *Macaca sylvanus:* Hesler and Fischer 2007; *Papio anubis*: Molesti et al. 2020), despite these being considered strong complementary markers of intentional production (Bates et al. 1975; Bruner 1981; Leavens et al. 2005) that may indicate a signaller is producing its communicative behaviour with a particular goal ‘in mind’ (Dennett 1983; Hobaiter and Byrne 2017; Byrne et al. 2017). This lack of data likely relates to the fact that most of the aforementioned studies have predominantly focused on monkey gesture use in experimental setups, in which the subjects were exclusively trained to produce one particular gesture type in one particular context to human experimenters. To date, only few monkey studies have focused on spontaneous and intraspecific intentional gesture production in non- experimental settings that may leave more room for observing flexibility in terms of goal persistence and means–ends dissociation (Maestripieri 1996; Hesler and Fischer 2007; Gupta and Sinha 2019; Molesti et al. 2020).

In this study, therefore, we particularly focussed on determining whether or not captive red-capped mangabeys (*Cercocebus torquatus*), a monkey species of the *cercopithecoid* superfamily of catarrhine primates that has been used as a model species in both vocal and gestural communication studies before (Maille et al. 2012; Bouchet et al. 2013; Aychet et al. 2020), made intentional and flexible use of gestures during their communicative interactions with conspecifics. We did this rigorously and from scratch, by assessing whether they showed *all* markers of intentionality during the spontaneous production of any of their potentially communicative body acts. A second aim was to find out whether the monkeys, if found to use intentionally produced gestures during their intraspecific interactions, also made use of free manual gesture types, i.e. gestures only produced with the hands, feet or limbs, without making contact with a substrate or partner. Given the fact that in previous studies red-capped mangabeys have been found to use trained manual gesture types in intentional ways towards human experimenters (Maille et al. 2012; Aychet et al. 2020), we expected the monkeys to be capable of intentional and manual gestural communication amongst conspecifics. The results of this study will contribute to a growing body of research towards the intentionality of monkey gestural communication that is needed for a more complete understanding of the spread of this capacity throughout the primate lineage and its potential implications for theories on human language origins.

## Methods

### Study site and subjects

We collected focal video footage from a total of 17 adult and subadult captive red-capped mangabeys living in three social groups at the Station Biologique de Paimpont, France (Table 1). Red-capped mangabeys are medium sized (7.5–10 kg) diurnal and predominantly terrestrial primates that naturally live in multimale-multifemale groups (Gautier-Hion et al. 1999; Jones and Sabater-Pi 1968; Cooke 2012; Orimaye 2017). Individual females and males are classified as adults when they are > 4 and 7 years of age, respectively, and as subadults when they are between 3 and 4 (females) or 7 (males) years of age, based on demographic data on a closely related species, i.e. grey-cheeked mangabeys (*Lophocebus albigena*; Chalmers 1968; Gautier-Hion and Gautier 1976; Deputte 1992).

**Table 1:** Social composition and individual details of the mangabeys in the study population

Group	Individual	Sex	Age at study (yrs) + (year of birth)
I	Chipie	Female	23 (1992)
	Many	Female	7 (2008)
	Chipse	Female	9 (2006)
	Tips	Male	4 (2011)
	Julie	Female	11 (2004)
	Gofrette	Female	19 (1996)
	Coet	Male	4 (2011)
	Maillette	Female	6 (2009)
II	Bell	Female	13 (2002)
	Kamel	Male	5 (2010)
	Zunie	Female	28 (1987)
	Joly	Female	15 (2000)
	Roby	Male	5 (2010)
III	Pirate	Male	23 (1992)
	George	Male	9 (2006)
	Carillon	Male	8 (2007)
	Elky	Male	6 (2009)

Each individual had access to one outdoor enclosure and one indoor enclosure with different sizes (ranging from 15.3 to 29 m^2^), which were connected by tunnels. The different enclosures for the groups were subdivided into several sub-enclosures separated by gratings. The monkeys could move freely between the inside and outside enclosures, except during keepers’ cleaning activities. The floor of the indoor enclosures was covered by straw and sawdust, whereas the floor of the outdoor enclosures was covered by bark or cement. Enrichment consisted of beams, ropes, chains, and tires. In the indoor enclosures, the temperature was stabilised at 22 ± 2 °C. The monkeys were fed twice daily with fresh vegetables and fruits in the morning and monkey chow in the afternoon. Fresh water was available ad libitum.

### Data collection

In February 2015, daily observations took place between 9 a.m. and 5 p.m. on a total of 13 observation days, with the monkeys being observed for a total of 4–6 h per day. Independent 15 min. continuous focal-animal sampling (Altmann 1974) was used to video record the behaviour of the 17 adult and subadult focal individuals for a total duration of 120 min per individual, using a Panasonic HC-X920 digital video camera. Focal animals were selected following a preset randomised order. Usually, the focus was on individuals from one group in the morning (between 9h:00 and 12h:00) and another in the afternoon (between 13h:00 and 17h:00), and the next day this order was alternated. If a focal subject moved outside the range of vision, the recording was stopped, but the recorded focal time was added to the total sampling time for that individual if it had lasted at least one minute. If a focal subject did not return within one minute after moving out of sight, the next session with a new focal animal was started. Comments were given on the general behaviour of both the focal individual and their potential recipient, their identity and sex, identities of other animals present within the same area of the enclosure, and whether or not an external entity appeared to be involved in the communicatory act. The video recordings were transferred to a PC and then loaded into Solomon Coder 15.1.13.0 for further video analyses.

### Identifying intentional gestural communication

In this study, the body acts that were further analysed for their potential to be defined as intentionally produced gestures comprised all instances of brachio-manual movements, leg movements, whole body movements, and body postures other than those performed during general locomotion, feeding, exploration, foraging and self-directed activities, and included those where non-mechanically effective physical contact with any other individual or a substrate was made.

To determine from the video recordings whether or not these body acts qualified as intentionally produced gestures, we first of all assessed whether they were ‘potentially communicative’. For this, we checked whether the monkeys were more likely to produce them in the presence rather than absence of other individuals (see Table 2 for operational criteria). For each single body act instance produced in the presence of other individuals, we subsequently determined whether the monkeys showed signs of audience checking and sensitivity to the attentional state of the recipient during their production (see Table 2 for operational criteria). To determine whether the production of these body acts was also associated with signs of goal-directedness, we additionally recorded for each body act instance whether or not the signallers showed signs of response waiting after producing it, which type of voluntary response was shown by the recipient, and whether or not the signaller showed any particular signs of goal persistence in case of an (apparently) unsatisfactory response from their recipient (see Table 2 for operational criteria). Recipients’ voluntary responses were scored as 1. ‘response’, i.e. a behaviour change that could occur in two forms: (a) recipient stops its current activity within a particular behavioural context and changes it to one within a different behavioural context, and (b) within one behavioural context the recipient changes its focus from self-directed behaviour or interacting with another individual than the signaller to interacting with the signaller, or 2. ‘no response’, i.e. the recipient does not show a behaviour change. Behavioural contexts identified were: grooming (including e.g. start/stop grooming, change of grooming position), agonistic (including aggression and submission), affiliative (social positive behaviours other than grooming), sexual (e.g. copulation, inspect or touch genitals), social play, and other (any behavioural context other than the aforementioned contexts, e.g. responding to keeper’s presence). Because a preliminary inspection of the monkeys’ interactions revealed that none of the signallers’ body acts concerned an external object or third party, the marker ‘gaze alternation’ was omitted from further analyses.

**Table 2:** Operational criteria used in this study to assess the intentionality of body act production (cf. Pika et al. 2003; Liebal et al. 2004; Leavens et al. 2005; Cartmill and Byrne 2007, 2010; Genty et al. 2009; Hobaiter and Byrne 2011; Roberts et al. 2014; Fröhlich et al 2016)

Criteria	Operationalisation
(i) Presence vs absence audience	At the time of the body act, the signaller was either (a) alone: signaller is the only individual present in the sub-enclosure and separated by at least 3 arm lengths from other individuals potentially present in adjacent sub-enclosures, or (b) social: signaller is present with other individuals in its sub-enclosure and/or other individuals are present within three arm lengths in an adjacent sub-enclosure
(ii) Audience checking	Within the 5 s. prior to body act production, the signaller was directly looking at the recipient, or the recipient was within the field of vision of the signaller who had its head oriented at most 45° either side from its looking direction straight ahead towards the recipient
(iii) Sensitivity to the visual attentional state of the recipient	Recipient was either (a) attending: within the 5 s prior to body act production, the recipient was directly looking towards the signaller, or the signaller was stationary within the field of vision of the recipient who had its head oriented at most 45°either side from its looking direction straight ahead towards the signaller, or (b) not attending: during the entire 5 s prior to body act production, the recipient’s head was turned away from the signaller and its attention was never directed towards the signaller, but distracted by other social partners or incidents in its environment
(iv) Response waiting	Following the end of body act production, the signaller paused signalling for at least 1 s and kept monitoring the recipient’s behaviour by maintaining visual contact
(v) Goal persistence	Following response waiting, it was scored for every instance of body act production whether the signaller (a) stopped producing further body acts, or (b) continued producing body acts, either by (i) repeating the same type of body act (*i.e.* ‘persistence’), *or* (ii) producing a new type of body act (i.e. ‘elaboration’). In case of (b), the process of assessing whether the marker of intentionality accompanied the body act production was restarted

### Identifying gesture types

Only those body act instances associated with all markers of intentionality (apart from gaze alternation) were considered intentionally produced gesture instances (see, e.g. Townsend et al. 2017; Ben Mocha and Burkart 2021). These included the instances where signallers stopped producing further body acts after receiving an apparently satisfying response from their recipient (i.e*.* an ‘intended’ behaviour change), as well as the instances where they continued producing body acts after receiving an apparently unsatisfactory response from their recipient (i.e. either no behaviour change at all or an ‘unintended’ behaviour change). Instances where the signallers did not produce any further body acts after receiving no behaviour change from their recipient were not included, as in this latter case it could not be assessed whether the signaller appeared to have had an initial goal or not (see, e.g. Townsend et al. 2017). All intentionally produced gesture instances were subsequently described in behavioural terms and classified as instances of a particular gesture type. These gesture types were then further categorised within one of the following gesture categories (Pika et al. 2003): (i) visual only gestures, i.e. distant signals representing movements of hands, body parts, or body postures, (ii) tactile gestures, i.e*.* visual signals that involve physical contact between the interacting animals (e.g. embrace), and (iii) audible gestures*,* i.e. visual signals that produce a sound during their production (e.g. a ground slap). Together, these gesture types represented the preliminary gestural repertoire of the monkeys in this study group. Using this preliminary gestural repertoire, we finally determined whether the mangabeys made use of ‘free’ manual gesture types as well, by checking which gesture types were produced by making movements of the arms and/or hands only, without making physical contact with a substrate or partner (de Waal 2003; Roberts et al. 2012, 2014). Note here that we decided to include all intentionally produced gesture types in the description of the monkeys’ preliminary gestural repertoire, also if they did not fulfil the often used requirement of having to have been used for an overall minimum of two times by at least two different individuals, or at least five times by one individual, to be included in the repertoire (e.g. Pika et al. 2003; Tomasello et al. 1985; Liebal et al. 2004; Hobaiter and Byrne 2011; Graham et al. 2016). This decision was made following a preliminary inspection of the gesture instances produced by non-focal individuals that were also caught on camera during focal video recordings, which revealed that the majority (85%) of gesture types produced by the focal individuals and reported in the preliminary gestural repertoire were also produced by other, non-focal, individuals in intentional ways (see Table 4 for details).

### Identifying flexibility of use

To assess the monkeys’ flexibility of gesture use in terms of means–ends dissociation, we determined the variety of behavioural contexts in which each particular gesture type was used. For this, the pre- and post-contextual information accompanying the signaller’s gesture production was analysed, with a focus on the ensuing behavioural change in the recipient or the immediately ensuing behavioural interaction between signaller and recipient following gesture production (Tomasello et al. 1997; Liebal et al. 2004). Depending on the nature of this assessment, we defined five contexts in which gestures were used: affiliative, agonistic, grooming, play, sexual. Instances of which the context remained ambiguous from the videos were coded as ‘unclear’.

### Inter-observer reliability

Inter-observer reliability coding of the occurrence of all intentionality criteria, as well as the classification of gesture types and context of use, was performed by a second observer for 25% of the data. For the intentionality criteria we used Cohen’s kappa (κ) to measure the degree of concordance between the two observers, and for gesture type and context we simply calculated the percentage of agreement. ‘Substantial’ to ‘excellent’ levels (McHugh 2012) of inter-observer agreement were found for all measured criteria (κ gesture category = 0.75, 89% agree; κ attentional state recipient = 0.74, 97% agree; κ audience checking = 1.0, 100% agree; κ response waiting = 0.90, 96% agree; κ change of recipient behaviour = 0.85, 95% agree; κ goal persistence = 0.72, 90% agree), as well as for gesture type (92% agree) and context of use (83% agree). The instances for which inconsistencies existed between observers were double checked and discussed between observers, after which it was decided to keep the original coder’s coding for all further instances, as these turned out to be scored most accurately.

### Statistics

We used R. 3.5.0 software (R Core Team 2018) to conduct statistical tests. Because of our relatively small sample size (*N* = 17 individuals), we mainly used descriptive statistics and non-parametric statistical tests. All tests were two tailed, with the alpha-level set at 0.05. The presence of each intentionality marker associated with the production of body acts was studied first at the level of body act occurrences (lumping all instances together), using tests for proportion comparisons (binomial and Chi Square tests), except for the first marker (presence vs absence audience), for which a ratio test was used. Then we also tested this at the individual level whenever possible, using Wilcoxon signed ranks tests, to account for potential inter-individual differences in signalling.

Regarding the marker ‘presence/absence audience’, we compared the monkeys’ rates of body act production between social vs alone conditions using a ratio test (Sahai and Kursid 1996; Martin and Austin 1996). Because of the small number of potentially communicative body acts produced in the alone condition, this was only possible on the group level. Regarding the marker ‘audience checking’, we assessed the likelihood of signallers’ body acts being associated with a signaller looking at the recipient prior to producing it. For the marker ‘sensitivity to the attentional state of the recipient’, we first tested whether or not body acts were preferentially produced in the presence of a visually attending or non-attending recipient. Moreover, we compared the proportion of each signal category (visual only or non-visual only, i.e. audible or tactile) depending on recipient’s attentional state. To run this analysis also at the individual level, we only kept the data of individuals that produced body acts both in the presence of attending and non-attending recipients (*N* = 7). Then, we tested the likelihood of body acts being associated with the marker ‘response waiting’. Regarding the marker ‘goal persistence’, we determined whether the form of goal persistence (stop signalling/repeat same signal/produce new signal) depended on the type of response by the recipient (response or no response). To run this test at the individual level as well, we only kept data of individuals that received both ‘responses’ and ‘no responses’ from their recipients (*N* = 13).

Finally, with regards to measuring means–ends dissociation, a preliminary inspection of our data revealed that the two hours of observation per individual provided insufficient data to address this question statistically. Therefore, we simply counted the number of different gesture types used within the same broad behavioural context, as well as the number of different contexts in which one particular gesture type was used (Pika et al. 2005b; Liebal et al. 2006; Call and Tomasello 2007; Genty et al. 2009). These were reported descriptively to get a first impression of the variety of contexts in which the particular gesture types were used.

## Results

### Intentionality of use

#### Presence/absence audience

From the 2040 min of focal observation (*N* = 17 individuals, 120 min per individual), the monkeys were observed to be ‘alone’ for a total of 132 min and ‘social’ for a total of 1908 min. In the social condition, the monkeys produced a total of 297 body acts not usually performed during general locomotion, feeding, foraging, exploration and self-directed activities (range = 1–40 per individual; median = 16), whereas in the alone condition they produced a total of five instances of such body acts. The monkeys were, thus, four times more likely to produce these body acts in the social condition compared to the alone condition (rate in social condition = 15.57; rate in alone condition = 3.788; RateRatio = 4.109; 95% CI = 1.70–9.90; *χ*^2^ = 11.7; exact mid-*p* = 0.00009).

Because the five instances of non-socially produced body acts were not potentially communicative to begin with, we only used the socially produced body acts (*N* = 297) to further assess whether their production was associated with each of the additional markers of intentional communication.

#### Audience checking

From the total of 297 instances of socially produced body acts, the majority (293; 99%) were preceded by audience checking (Binomial test: *N* = 297, *P*_exp_ = 0.50, *P*_obs_ = 0.99, *p* < 0.001). This effect was also found at the individual level. All 17 individuals showed audience checking, with the mangabeys visually orienting to their recipient before producing their behaviour in the majority of cases (Wilcoxon signed rank test: *N* = 17, *V* = 153, *p* < 0.001).

#### Sensitivity to the attentional state of the recipient

For two out of the 297 socially produced body act instances, the attentional state of the recipients could not be determined due to insufficient recording conditions. From the remaining 295 instances, the majority (280; 95%) were produced by the signallers when recipients were visually attending (Binomial test: *N* = 295, *P*_exp_ = 0.50, *P*_obs_ = 0.95, *p* < 0.001). This effect was also found at the individual level. All 17 individuals showed a sensitivity to the attentional state of their recipient, with the mangabeys generally producing body acts more often in front of visually attending compared to non-visually attending recipients (Wilcoxon signed rank test: *N* = 17, *V* = 136, *p* < 0.001).

**Fig. 1:**
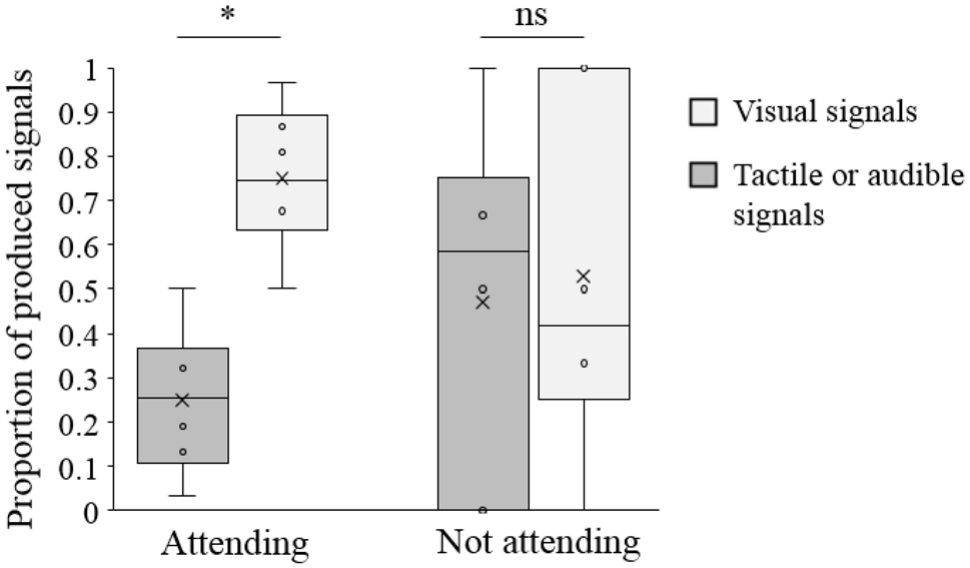
Proportions of signallers’ use of visual signals and non-visual signals (tactile or audible signals) in relation to the visual attentional state of their recipients (Wilcoxon signed rank tests: *p ≤ 0.050; ns: *p* > 0.050, *N* = 7 individuals)

From the 280 instances produced to visually attending recipients, 234 (84%) were visual only, 42 (15%) were tactile and four (1%) were audible. From the 15 body act instances that were produced when recipients were not visually attending, six (40%) were visual only, eight (53%) were tactile and one (7%) was audible. Further analysis of these results showed that there was a significant association between the attentional state of the recipient and the signal category used by the signallers. Signallers used visual signals less often and non-visual signals more often than expected by chance when recipients were not visually attending (*χ*^2^ (1, *N* = 295) = 17.82; *p* < 0.001), despite some variation in the use of gesture categories to non-visually attending recipients at both the group level (Binomial test: *N* = 15, *P*_exp_ = 0.50, *P*_obs_ = 0.40, *p* = 0.607) and the individual level (Fig. 1, Wilcoxon signed rank test: *N* = 7, *V* = 12, *p* = 0.829).

#### Response waiting

From the total of 297 socially produced body act instances, the majority (285; 96%) were followed by response waiting (Binomial test: *N* = 297, *P*_exp_ = 0.50, *P*_obs_ = 0.96, *p* < 0.001). This effect was also found at the individual level. All 17 individuals showed response waiting after gesture production, with the mangabeys monitoring a recipient’s behaviour after producing body signals in the majority of cases (Wilcoxon signed rank test: *N* = 17, *V* = 153, *p* < 0.001).

#### Goal persistence

A total of 104 (35%) out of all 297 socially produced body act instances resulted in ‘no response’ from the recipients (i.e. no behaviour change at all) and 193 (65%) out of the 297 instances led to a response (i.e. a behaviour change). From the 104 instances leading to no response, signallers stopped their body act production in 50 (48%) cases and continued producing further body acts in 54 (52%) cases. From the 193 instances leading to a response, the signallers stopped further body act production in 127 cases (66%) and continued with further body act production in 66 cases (34%).

**Fig. 2:**
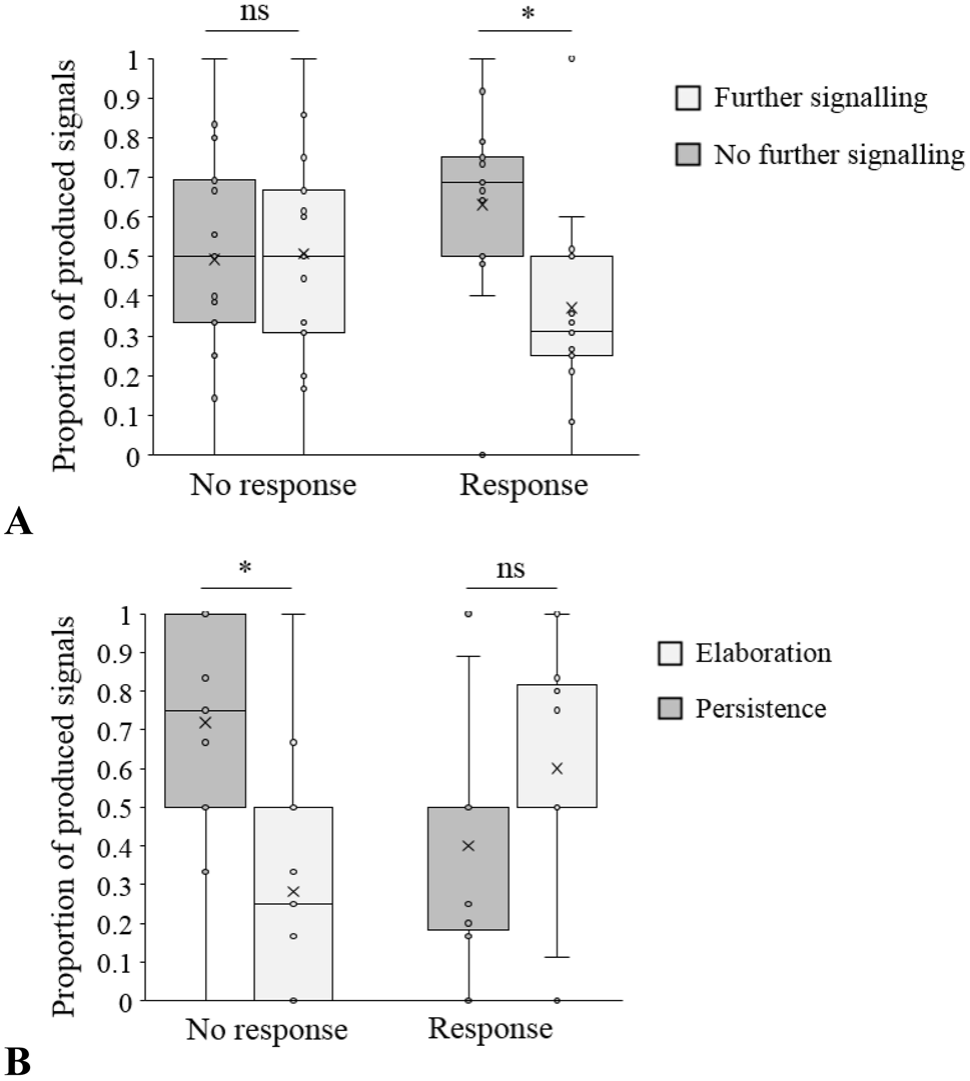
**A** Proportions of further body act production (‘further signalling’) or no further body act production (‘no further signalling’) by signallers in relation to receiving either a response or no response from their recipient (*N* = 15 individuals). **B** Proportions of elaboration or persistence as further signalling behaviour by signallers in relation to receiving a response or no response from their recipient (*N* = 13 individuals). Wilcoxon signed rank tests: *p ≤ 0.050; ns: *p* > 0.050

In case of an apparently unsatisfactory response from the recipient (i.e. either a non-intended behaviour change or no behaviour change at all), 13 of the 17 individual signallers showed further body act production in the form of persistence and eight of the 17 individual signallers showed further body act production in the form of elaboration. The form of this further signalling behaviour was contingent on the type of unsatisfactory response they had received from their recipients. When the monkeys continued further body act production after receiving no behaviour change at all (*N* = 54), they persisted with the same type of body act behaviour in 39 cases (72%) and elaborated their initial body act behaviour by using a different type in 15 cases (28%). If they continued further body act production after receiving an apparently non-intended behaviour change (*N* = 66), they elaborated their body act behaviour in 38 cases (58%) and persisted with the same type in 28 cases (42%). Further analysis of these results showed that there was a significant association between the type of recipient response and the signaller’s production of further body acts. The monkeys were more likely than expected by chance to stop producing further body acts after having received a response from their recipient compared to having received no response (*N* = 297, *χ*^2^ = 8.8191, *df* = 1, *p* value = 0.003), and to repeat the same body act behaviour (persist) as further signalling behaviour in cases where they received no response from their recipient compared to having received an apparently wrong response; in the latter case they also often decided to elaborate their communicative behaviour by using a different body act (*N* = 120, *χ*^2^ = 10.694, *df* = 1, *p* value = 0.001). The same effect was also found at the individual level (Fig. 2A and B, Wilcoxon signed rank test: persistence after response: *N* = 13, *V* = 24, *p* = 0.438; persistence after no response: *N* = 13, *V* = 10, *p* = 0.041).

In total, 243 out of the 297 socially produced body act instances (82%) were each associated with all the aforementioned markers of intentionality (see Table 4) and classified as intentionally produced gesture instances. These were further used to describe the different gesture types produced by these monkeys and, by extension, the preliminary gestural repertoire of this study population.

### Description of gesture types

From the total of 243 intentionally produced gesture instances, we identified 20 different gesture types (Table 3). These were all produced at least once by at least one individual in concordance with all markers of intentionality (Table 4). Moreover, for all gesture types except ‘roll on ground’, ‘salto’, and ‘swing body’ there were observed cases of at least one additional non-focal individual producing this gesture type intentionally as well (Table 4). One other observed body act, ‘shake object’, was not accompanied by all markers of intentionality and was also not produced by any other non-focal individual. This body act was, therefore, not included as a gesture type in the preliminary gestural repertoire (Table 4).

**Table 3:** Overview and description of the different gesture types produced by red-capped mangabeys during this study

Gesture name	Description
Visual gestures	
1. Crouch	Subject is in crouching position on the ground in front of the other, belly touching the ground, on four legs
2. Head bob	Subject makes short forward movement of the head, ‘lunging’ its head towards the other, while staring at the other intently
3. Head shake	Subject moves head in quick, jerky movements in front of the other, horizontally and/or vertically
4. Hop	Subject jumps/ hops in the air with both feet leaving the ground, either in one position or moving around in front of the other
5. Lie down wiggling	Subject is lying down on its belly in front of the other, wiggling its body at the same time. This can also occur with one arm extended towards the other, with body and arm wiggling at the same time
6. Make grabbing movement towards*	Subject throws arm towards the other with hand closing at the end of the movement
7. Move arm in front*	Subject puts its arm with elbow flexed in 90° in front of its face or its body
8. Present body part	Subject exposes one particular body part to the other, by orienting its body appropriately. Subject can present different body parts (including back, belly, rear, neck, penis, head, side) using different postures. This occurs in three main positions: while seated, standing, or lying down rigidly (subject is lying down on the belly or on the back in front of the other, not moving, exposing body part). At least 25 different varieties of this signal were recorded (*e.g*. present back seated arms relaxed, present back seated one arm up, present back seated two arms up, present back lying on belly, etc.)
9. Roll on ground	Subject rolls on the ground in front of the other
10. Somersault	Subject jumps and makes a forwards or backwards salto/flip
11. Swing body	Subject moves its body in quick movements in front of other: swinging its body from side to side, seated, arms relaxed
12. Throw arm towards*	Subject extends and throws arm (arm extended) with hand open, palm facing down, towards the other in a brief, quick movement
13. Throw body towards	Subject throws its upper body in direction of other, with two hands and arms stiff, thereby hitting the ground or support
14. Throw hand towards (hand fling)*	Subject ‘throws’ one hand towards the other in a brief movement, with hand open, palm facing down, no movement of arm
Tactile gestures	
15. Embrace	Subject puts one arm or both arms around the body of the other, dorsally (by the back) or ventrally (by the front)
16. Grab body part	Subject grabs a body part of the other with its hand or feet, without pulling body part. The subject can hold the body part briefly (< 1 s) or longer (> 1 s). Body parts include: top of the head, arm, hand, penis, tail, hips
17. Hit/slap body part	Subject hits a body part of the other with a flat hand, in a quick movement (< 1 s). Body parts include: top of the head, limb, back, hand, tail
18. Put body part on other	Subject places its hands or feet gently on a body part of other (> 1 s). Body parts of others include tail, arm, top of the head, back
Audible gestures	
19. Kick object	Subject jumps in the air and stamps once on an object (e.g. cage) with two feet
20. Shake object	Subject holds and shakes an object or other support (cage) with its hands or with its hand and feet
21. Slap object	Subject hits/slaps the ground or an object (e.g. cage) with one or two hands

Gesture types are further specified per signal category (visual only, tactile, or audible signals). Manual gestures are indicated with *

**Table 4:** Intentional body acts per individual. Indicated per signal type is how many instances (*x*) of a signallers’ total body acts of this type (/*y*) adhered to all of the following intentionality markers: audience checking, attentional state adjustment, response waiting, and goal persistence

Focal ID	Sex	Signal type																					Total number of intentional gesture types per ID
		Visual														Tactile				Audible			
		Crouch	Head bob	Head shake	Hop	Lie down wiggling	Make grabbing movement towards**	Move arm in front**	Present body part	Roll on ground	Salto	Swing body	Throw arm towards**	Throw body towards	Throw hand towards (hand fling)**	Embrace	Grab body part	Hit/slap body part	Put body part on other	Kick object	Shake object	Slap object	
Chipie	F						1/1		26/31^− # *^														2
Many	F								29/33^− +*^				1/1								0/1^*^		3
Chipse	F		1/1						4/9^+ *^				2/2		2/2			1/1		1/1			6
Tips	M					1/1			4/5^−^				5/5				1/1	2/3^*^	1/1				6
Julie	F								1/2^*^														1
Gofrette	F							2/2	5/5														2
Coet	M			1/1			0/1^*^		7/9^+ *^				5/6^*^			1/1	2/2		2/3^*^				6
Mailette	F								5/6^*^								0/1^*^						1
Belle	F								1/2^−^										1/1				2
Kamel	M				1/1	2/2	1/1		3/4^*^				8/10^*^		0/1^*^	3/4^#^	1/1	4/5^# *^	1/2^*^				9
Zunie	F				1/1		1/1		10/11^*^				2/3^*^		2/2			1/1					6
Joly	F								6/7^*^								0/1^*^						1
Roby	M				3/3		1/1	1/1	17/20^*^	1/1	1/1						1/1						7
Pirate	M						1/1		12/13^*^				6/6	1/1									4
George	M								1/4^+ *^										2/3^# *^			1/1	3
Carillon	M																	1/1					1
Elky	M	1/2^*^		1/1	4/5^*^	1/1	0/1^#*^		6/6			1/1	5/8^# *^			3/3	2/2	5/7^# *^		1/1		1/2^*^	12
Total nb		1/2	1/1	2/2	9/10	4/4	5/7	3/3	137/167	1/1	1/1	1/1	34/41	1/1	4/5	7/8	7/9	14/18	7/10	2/2	0/1	2/3	**243/297**
Total by focal ID		1/1	1/1	2/2	4/4	3/3	5/7	2/2	16/16	1/1	1/1	1/1	8/8	1/1	2/3	3/3	4/6	6/6	5/5	2/2	0/1	2/2	
Additional non-focal: nr ID (+ #)		1 (1)	1 (1)	1 (1)	2 (2)	1 (3)	2 (2)	0	n.a	0	0	0	6 (16)	2 (12)	3 (9)	2 (4)	4 (10)	7 (15)	2 (2)	0	0	2 (3)	

Where *x* < *y*, a total of (*y* − *x*) body acts were either (−) not preceded by audience checking; (+) not adjusted to attentional state of recipient; (#) not followed by response waiting, and/or (*) not associated with markers of goal persistence. Body acts not associated with all mentioned markers of intentionality were not considered intentionally produced gesture instances. Also indicated are the observed instances of particular gesture use by non-focal individuals (number of additional non-focal ID producing the gesture + total number of observed instances, #)

*ID* individual, *F* female, *M* male

**Manual gesture type

Four of the 20 gesture types were categorised as ‘tactile’ gestures, two were categorised as ‘audible’ gestures, and 14 were categorised as ‘visual only’ gestures. Of the 14 visual only gesture types, four were ‘free’ manual gesture types (gesture types 6, 7, 12 and 14 in Table 3), i.e. produced by making movements of the arms and/or hands only, without making physical contact with a substrate or partner (sensu de Waal 2003; Roberts et al. 2012, 2014).

### Flexibility of use

With regards to flexibility of gesture use in terms of ‘means–ends dissociation’, we described the different contexts in which the different gesture types were used across all individuals (Table 5). Two manual gesture types (‘throw arm towards’ and ‘throw hand towards’) and three non-manual gesture types (‘hit body part’, ‘put body part on other’ and ‘present body part’) were used flexibly, i.e. in both the social play context and at least one additional behavioural context (i.e. agonism or grooming, with ‘present body part’ being produced in three contexts additional to play, i.e. groom, affiliative and sexual). In addition, the monkeys made use of several different gesture types within the same functional context for both the play context (13 different gesture types used) and agonistic context (nine different gesture types used).

**Table 5:** Social contexts of gesture production

Signal categories	Gesture type	Context						Total
		Agonistic	Affiliative	Grooming	Play	Sexual	Unclear	
Visual	Crouch	0	0	0	0	0	1	1
	Head bob	1	0	0	0	0	0	1
	Head shake	0	0	0	2	0	0	2
	Hop	0	0	0	8	0	1	9
	Lie down wiggling	0	0	0	3	0	1	4
	Make grabbing movement towards*	4	0	0	0	0	1	5
	Move arm in front*	3	0	0	0	0	0	3
	Present body part	13	1	53	10	38	22	137
	Roll on ground	0	0	0	1	0	0	1
	Somersault	0	0	0	1	0	0	1
	Swing body	0	0	0	1	0	0	1
	Throw arm towards*	18	0	0	8	0	8	34
	Throw body	1	0	0	0	0	0	1
	Throw hand towards*	3	0	0	1	0	0	4
Tactile	Embrace	0	0	0	6	0	1	7
	Grab body part	0	0	0	6	0	1	7
	Hit/slap body part	4	0	0	8	0	2	14
	Put body part on other	2	0	0	1	0	4	7
Audible	Kick object	0	0	0	0	0	2	2
	Slap object	1	0	0	0	0	1	2
Total		50	1	53	56	38	45	243

Free manual gesture types are indicated with *

## Discussion

### Intentionality of gesture use

In this study, we investigated whether the intraspecific production of potentially communicative body acts of a group of captive red-capped mangabeys qualified as flexible and intentional gestural communication, and whether individuals used ‘free’ manual gestures during their communicative exchanges as well. The mangabeys produced a total of 21 types of body acts that were not usually produced as more general locomotion, foraging, exploration and self-directed activities. These were all mechanically ineffective and led to a voluntary response by recipients. Of these, 20 were classified as intentionally produced gesture types, as their production fulfilled specific markers characterising intentional communication. These gesture types were generally (i) produced in the presence of recipients, (ii) preceded by audience checking, (iii) adjusted to the attentional state of the recipient, (iv) followed by response waiting, and (v) accompanied by signs of goal persistence. More specifically, in terms of attentional state adjustment, the mangabeys used visual only gesture types more frequently in situations with visually attentive recipients, and tactile and audible gesture types in situations with non-visually attentive recipients. Concerning goal persistence, they often continued producing further body acts if their initial behaviour was followed by an apparently unsatisfactory response from the recipient. Red-capped mangabeys, thus, appear able to communicate in intentionally communicative and goal-directed ways with their conspecifics.

These results corroborate findings of two recent studies providing evidence for intraspecific intentional gesture production in two other catarrhine monkey species, wild bonnet macaques (Gupta and Sinha 2019) and captive olive baboons (Molesti et al. 2020). The study on the bonnet macaques paid special attention to the markers response waiting and goal persistence during gestural interactions between already attentive communicative partners, while the study on the olive baboons examined the markers audience checking, attentional state adjustment, and response waiting. Our study adds to the growing body of evidence that first-order intentional communication may be a more universal pattern in primate communication, by providing an assessment of the gestural communication of a single group of monkeys with regards to *all* markers of intentionality. Due to this choice of markers, it conquers recent debates concerning the validity of using only one or a restricted set of markers when assessing animals’ intentional communication skills (see, e.g. Liebal et al. 2013; Townsend et al. 2017; Graham et al. 2020; Ben Mocha and Burkart 2021; Rodrigues et al. 2021). The different markers of intentionality jointly assess several aspects of both the communicative and goal-directed properties of socially produced behaviour, with each marker contributing its own added value to this assessment (Ben Mocha and Burkart 2021). Ideally, therefore, any study claiming that an animal’s apparently communicative behaviour is ‘intentional’ should clearly account for both the communicative and goal-directed aspects of this behaviour, by providing converging evidence for the use of as many markers of intentionality as possible (Liebal et al. 2013; Townsend et al. 2017; Graham et al. 2020; Ben Mocha and Burkhart 2021) and providing as much detail as possible on their frequency and distribution of use (Rodrigues et al. 2021; Ben Mocha and Burkart 2021).

According to several scholars (e.g. Bard 1992; Leavens et al. 1996; Bard et al. 2014), this includes assessing the use of gaze alternation accompanying signal production that is directed towards an external entity, as this could provide evidence that an animal is capable of communicating ‘about’ something to its recipient. Yet, such triadic communicative interactions are rare to observe in natural settings, as the majority of communicative interactions across adult primates occur as dyadic events, in which signallers merely aim to direct the behaviour of their recipients relative to their own social motivations and goals (Hurford 2007; Liebal et al. 2013; Tomasello and Call. 2019). Consequently, this is the one marker that is most often missing or omitted in studies towards intentional communication amongst conspecifics. Likewise, in our study, we only observed one instance of potential triadic communication in the food begging context, with the video angle being insufficient to reliably code the signaller’s use of gaze alternation. Because of this relatively rare chance of observing interactions involving an external entity in naturally occurring communicative events, a more common way to study this in primates has been by using experimental food requesting paradigms, in which the subjects are confronted with an out of reach food item in the presence of a human experimenter (e.g. Leavens et al. 2004). Such studies revealed that ape (Bard 1992; Leavens and Hopkins 1998, Leavens et al. 2004; Lucca et al. 2018) but also some monkey species (e.g. squirrel monkeys: Anderson et al. 2010; olive baboons: Meunier et al. 2013; Tonkean macaques: Canteloup et al. 2015a) direct their (trained) begging gestures towards a food item in conjunction with showing gaze alternation between the experimenter and the food. Such observations are usually taken to suggest that the study subjects are capable of communicating about the food item to the experimenter in a (proto-) imperative manner (e.g. Bard 1992), i.e. with the aim of requesting the experimenter’s help to provide it to them. Although the debate about the psychological implications of such findings is still ongoing (e.g. Tomasello and Call 2019; Leavens 2021), from a philosophical point of view they imply that the signallers have an understanding of others as causal agents, i.e. they show first-order intentionality (Dennett 1983; Hurford 2007). A similar experimental setup was recently used in the same captive population of red-capped mangabeys as investigated in our study (Aychet et al. 2020). The study showed that the individuals of our study group were capable of this communicative capacity as well. In combination with the use of all the other behavioural markers of intentional communication during intraspecific communicative interactions (this study), the communication observed in this group of captive red-capped mangabeys is therefore remarkably similar to that found in the apes and can be classified as first-order intentional communication.

### Flexibility of gesture use

In terms of ‘means–ends dissociation’ (Bruner 1981), we found that the monkeys produced a quarter of their currently described gestural repertoire in more than one behavioural context. Furthermore, in both the social play and agonistic context the mangabeys produced several different gesture types (but see Pika and Deschner 2019 for a critical view on context assessment). These results suggest that the individuals studied here may use the same signal for different ends and different signals for the same end interchangeably, a capacity shared with the apes (e.g. Pika et al. 2005b; Liebal et al. 2006; Genty et al. 2009) as well as some other monkey species (pigtail macaques: Maestripieri 1996; Barbary macaques: Hesler and Fischer 2007; olive baboons: Molesti et al. 2020). It should be noted, however, that the proportion of gesture types used flexibly across contexts in our study group appears relatively low, i.e. not only when compared with the apes but also when compared with the other monkey species showing this form of communicative flexibility. There may be several explanations for this. One explanation is that the individual observation times in our study were probably too short to enable a complete understanding of the entire gestural repertoire and all its uses in our study population (see, e.g. Hobaiter and Byrne 2011). Indeed, a preliminary inspection of opportunistically recorded non-focal gesture use in the current study revealed that both the repertoire size and the proportion of gesture types used flexibly may be more extensive in this population than currently reported. Furthermore, differences in the observed degree of flexibility across study species and study populations may be due to the use of different definitions for gesture and different levels of detail used to describe gesture types and production contexts (e.g. Hobaiter and Byrne 2017; Molesti et al. 2020; Rodrigues et al. 2021). For instance, in the current study facial expressions were not considered gestures (see Byrne et al. 2017 for argumentation to classify facial expressions and gestures as independent systems), while in other monkey studies investigating intraspecific gesture use facial expressions were included in the analyses. Defining facial expressions as gestures inevitably leads to a larger repertoire size and, arguably, to a higher potential for flexible use. And another methodological issue to keep in mind is that differences in observed levels of flexibility across study groups may correspond to differences in group compositions and rearing environments (reviewed in Rodrigues et al. 2021). For example, when young individuals are over-represented in a group, higher levels of means–ends dissociation may be observed simply because young individuals tend to play more often than older individuals (e.g. Fröhlich et al. 2016), and play is characterised by a diversity of behavioural patterns from a variety of contexts (Yanagi and Berman 2014; Byrne 2016). From an ultimate perspective, an alternative explanation for the observed species differences in the level of means–ends dissociation could be that red-capped mangabeys have simply not developed a high degree of communicative flexibility in the visual domain. Although future studies with higher sample sizes, longer observation periods and uniform sampling methods are crucially needed to make valid species comparisons (Rodrigues et al. 2021), such a lack would be interesting, as it could reveal more about the selection pressures linked to the evolution of communicative complexity in relation to the specific socio-ecological environments that animals are reared in (e.g. ‘social complexity hypothesis for communication’: Freeberg et al. 2012; see also McComb and Semple 2005; Cartmill and Maestripieri 2012; Bard and Leavens 2014; Fröhlich and Hobaiter 2018; Peckre et al. 2019; Leavens et al. 2019; Rebout et al. 2020). Obtaining more insight into the differences and similarities of communicative capacities from a multitude of carefully chosen captive and wild representatives of the more than 50 genera of primates could therefore lead to a better understanding of the selection pressures that may have driven their origins, and, ultimately, the evolution of increasingly complex forms of intentional communication, including language (Leavens et al. 2019; Rodrigues et al. 2021).

### Flexibility of goal persistence strategies

Another way of investigating flexibility of gesture use is by assessing a signallers’ use of goal persistence strategies in cases of communicative failure (Cartmill and Byrne 2007; 2010; Roberts et al. 2013). In the present study, we found a significant association between the type of further signalling behaviour by signallers and the type of unsatisfactory response given by their recipients. Persistence was the most frequent form of continuation when recipients did not respond with a behaviour change at all. Yet, in cases where recipients responded with an (apparently) ‘wrong’ behaviour change to the initial gesture, they did not show this preference for persistence, and often used elaboration as a form of further signalling behaviour as well. From a cognitive perspective, such a differentiation in communicative response characteristics to different forms of unsatisfactory responses from recipients could indicate that the red capped mangabey signallers, similarly to the apes (Cartmill and Byrne 2007; Leavens et al. 2005; Roberts et al. 2013; Genty et al. 2015), are capable of understanding different degrees of recipient comprehension, and use different types of repair strategies in response to this. In the ape studies, such findings are usually taken to suggest that the signallers are able to take into account the basic mental states of their recipients (Genty et al. 2015). Based on the results from the present study, in which the mangabeys behave very similar to apes in this specific domain of communicative complexity, the mangabeys might therefore be considered capable of basic mental state attribution as well. Notably, however, in a recent experimental study (Aychet et al. 2020) in which the same study population of red-capped mangabeys was subjected to an experimental design comparable to that generally used to assess a signaller’s sensitivity to recipient comprehension in ape studies, these monkeys showed a different response. In the study, the animals were trained to use begging gestures to request help from a human experimenter to access an out of reach food reward, with the experimenter showing different levels of responsiveness. The results from that study showed that the animals persisted using the same begging gesture in cases of receiving no response at all from the experimenter. Critically, however, they did not show signs of elaboration in cases of receiving the ‘wrong response’ from the experimenter. Although these results may reflect real interspecific differences with the apes, who have been found to show more flexibility in comparable experimental contexts (Aychet et al. 2020), the fact that in the current study we found more diverse response characteristics to apparent misunderstandings from recipients in intraspecific communicative interactions could indicate that these monkeys are actually more flexible in their communicative interactions than granted by experimental studies alone. In the experimental food begging context, the animals had been trained to use only one particular gesture type and may simply not have had an alternative ‘at hand’ when the communication failed in this novel context. These observed response differences across different study settings contribute to recent debates related to the question to what extent differences in testing conditions affect findings of gesture use across study populations (Bourjade et al. 2015; Leavens et al. 2019; Rodrigues et al. 2021). Moreover, they reiterate the importance of obtaining more data from both inter- and intraspecific communicative interactions from a more diverse range of species in both experimental and natural settings, to gain a better understanding of animals’ communicative capacities from a broader perspective (Rodrigues et al. 2021).

### Manual gesture use

Finally, with regard to manual gesture production, we found that the monkeys made use of four ‘free’ manual gesture types. These were produced in variable frequencies divided over 11 of the 17 focal individuals. It has been suggested that the capacity for intentional and flexible manual gesture production formed the starting point of human language evolution, and, as manual gesture types were argued to be virtually non-existent in monkey species, that this starting point of human language evolution lies in the hominoid lineage (Call and Tomasello 2007; de Waal 2003, 2016; Pollick and de Waal 2007; de Waal and Pollick 2011; Cartmill and Maestripieri 2012; Roberts et al. 2012, 2014; Tomasello and Call 2019). The results from our study contradict this idea. The mere fact that the red capped mangabeys produced four free manual gesture types shows that intentionally produced manual gesture types are existent in monkey gestural repertoires. However, before further species comparisons can be made, systematic future studies assessing the proportion of manual gesture types in monkey species’ gesture repertoires, as well as their flexibility of use, are first needed. Based on our current findings, the hypothesis is that the proportion of manual gesture types in the gestural repertoire of red capped mangabeys will be comparable to that found in other monkey species living in similarly complex social societies (Hesler and Fischer 2007; Gupta and Sinha, 2019; Molesti et al. 2020), but will be considerably lower to that found in the apes (Genty et al. 2009; Hobaiter and Byrne 2011; Roberts et al. 2012, 2014; Graham et al. 2016; Knox et al. 2019). As mentioned, the assessment of the red capped mangabeys’ gestural repertoire is not complete yet and needs to be further investigated before further conclusions can be drawn about this.

Based on the results of our study, it currently seems that concerning flexibility and manual gesture use the apes outperform monkeys, although these capacities are not absent in monkey species investigated so far. Further comparative investigations of means–end dissociation, goal persistence and manual gesture use from a more diverse range of species are crucial to obtain a more complete view on these capacities in the primate lineage. This will reveal whether these capacities indeed turn out to be some of the key differences between monkey and ape communicative capacities (e.g. Cartmill and Maestripieri 2012), in addition to apes’ alleged capacities for higher order forms of intentionality underlying their communicative behaviour (e.g. Crockford et al. 2017) and turn-taking abilities (Pika et al. 2018). Yet, to unravel these differences it is important that uniform definitions and methodologies are used across studies. Investigating this further in future endeavours will shed more light on the presence of these capacities in other species than the apes, which can ultimately be used to better understand how human language evolved.

In sum, in this study, we provide systematic evidence that the captive population of red-capped mangabeys residing at the Station Biologique in Paimpoint use their naturally occurring body acts in flexible and intentional communicative ways to communicate with conspecifics. Specifically, their gestural production was characterised by all intentionality markers, thereby expanding previous studies that only tested subsets of markers of intentionality. The preliminary intentional gestural repertoire of our study population includes body postures, but also free movements of the hands and arms. They produce their gestures flexibly, both in terms of behaviour (e.g. elaboration during goal persistence) and context. In addition, they have been shown to engage in potential triadic gestural communication in an experimental food requesting paradigm (Aychet et al. 2020). In combination with the fact that studies on the vocal communication of these monkeys have indicated their capacity for syntactic like semantic vocal communication (Bouchet et al. 2010), we argue that red-capped mangabeys are a valuable species to consider for further comparative communication studies. The results of our study contradict claims that the starting point for flexible and intentional manual communicative strategies appeared in the hominoid lineage. In stark contrast, and in concordance with recent findings in bonnet macaques and olive baboons (Gupta and Sinha 2019, Molesti et al. 2020), our results show that some species belonging to the monkey lineage of catarrhine primates show these capacities as well. The difference between monkeys and apes may, therefore, be in degree, rather than in kind. Investigating which selection pressures have driven these differences in degree and have led to the highly complex forms of intentionality needed for ostensive communication found in human language are now needed to move the debate on human language origins to the next level.

## Data Availability

On request.

## Code availability

On request.
